# Novel formulation of neutral lactase improves digestion of dairy products in case of lactose intolerance

**DOI:** 10.1186/2045-7022-1-S1-P104

**Published:** 2011-08-12

**Authors:** Lucas Fraissl, Roland Leitner, Albert Missbichler

**Affiliations:** 1 University of applied Sciences Wr. Neustadt, Dept of Biotechnical Processes, Tulln, Austria; 2 Medical University of Vienna, Austria, Dept. of Medical Biochemistry, Vienna, Austria; 3 Sciotec Diagnostic Technologies GmbH, Tulln, Austria

## Background

Lactose intolerance is the insufficient ability to digest lactose, a sugar commonly found in dairy products. It is caused by a deficiency of the enzyme lactase, which is resident in the small intestine. Lactase breaks down lactose into glucose and galactose, which are readily absorbed into the bloodstream. Lactase deficiency develops slowly over time, most persons concerned do not experience symptoms of lactose intolerance until late adolescence or adulthood. Typical symptoms are abdominal pain, bloating, diarrhea and nausea.

## Treatment

Most people with lactose intolerance can tolerate some amount of lactose in their diet. Thus a reduction of dairy products is the method of choice. Nevertheless, lactose often is present in processed food and instant food, making it difficult to keep a lactose-reduced diet. Lactase, the enzyme degrading lactose, was made available as a food supplement by different companies. All these products use acid lactase in a formulation that makes enzyme activity available in the stomach. Depending on food intake, activity of acid lactase is destroyed within 15 to 45 min in the stomach by gastric juice. Thus it is difficult for the user to find a dosage of enzyme that reliably degrades the lactose taken up with food.

## Improvement

The new formulation presented here for the first time uses neutral lactase, produced in klyveromyces lactis, a yeast well known from cheese production. The enzyme is stabilized in small pellets of 1 mm diameter, which are enteric coated with shellac thus protecting lactase from gastric juice. Because of the small size the pellets pass the stomach within 15 min to reach the small intestine. In the neutral surroundings the pellets disintegrate and release the activity of the neutral lactase. Because of the slow peristalsis in the small intestine neutral lactase activity persists for approximately 4 hours. This long period of activity ensures reliable and complete degradation of lactose in the small intestine.

## Results

An observational study with 64 persons showed high acceptance of the product and a highly significant reduction of symptoms: abdominal pain: 58%; bloating: 75%; diarrhea 67%; nausea 58%.

